# How Does Shyness Affect Chinese College Students' Tendency to Mobile Phone Addiction? Testing the Mediating Roles of Social Anxiety and Self-Control

**DOI:** 10.3389/fpubh.2022.902425

**Published:** 2022-07-13

**Authors:** Xinwei Li, Weijian Li, Mengxian Liu, Weilong Xiao, Hui Zhou

**Affiliations:** ^1^College of Teacher Education, Zhejiang Normal University, Jinhua, China; ^2^Key Laboratory of Intelligent Education Technology and Application of Zhejiang Province, Zhejiang Normal University, Jinhua, China; ^3^School of Electronic Commerce, Zhejiang Business College, Hangzhou, China; ^4^Jinhua Advanced Research Institute, Jinhua, China

**Keywords:** college students, shyness, social anxiety, self-control, mobile phone addiction tendency

## Abstract

**Background and Aims:**

Mobile phone addiction among college students has gained considerable research attention because of its adverse effects on their health and academic performance. However, little is known about the mechanisms underlying the relationship between shyness and mobile phone addiction among college students.

**Methods:**

Four questionnaires were used to examine whether mobile phone addiction tendency was predicted by shyness and the mediating roles of social anxiety and self-control among 3,189 Chinese college students. Correlation and mediation analyses were conducted using Hayes PROCESS.

**Results:**

The results showed that (1) social anxiety (indirect effect = 0.22, 95% CI = 0.18–0.26) and self-control (indirect effect = 0.23, 95% CI = 0.21–0.25) played a partial mediating role in the relationship between shyness and mobile phone addiction tendency; (2) social anxiety and self-control also mediated the link between shyness and mobile phone addiction tendency sequentially (indirect effect = 0.10, 95% CI = 0.09 to 0.12).

**Conclusion:**

These results suggest that mobile phone addiction among shy college students could be eliminated by alleviating social anxiety and strengthening self-control.

## Introduction

Adolescents and youth are major forces that will make the world better in the future. However, they face serious problems, such as mobile phone addiction. Globally, there is a high incidence of mobile phone addiction among adolescents (1). One survey showed that the prevalence of problematic mobile phone use or addiction is between 10% and 31% in British and Korean adolescents, respectively (2). In China, the prevalence of problematic phone use ranges between 15 and 30% (3). Mobile phone addiction/excessive usage of mobile phones could lead to depression (4), academic difficulties (5), and poor creativity and productivity (6, 7). Therefore, understanding the mechanisms underlying mobile phone addiction may be significant for college students who are trying to quit mobile phone addiction.

Shyness is the tendency to feel nervous, worried, or embarrassed in the presence of others because of the fear of feeling judged by others in interpersonal interactions (8, 9). In previous research, there were two types of studies: First, some studies distinguish shyness when an individual's score on the scale exceeds a certain point, and the individual could be considered shy (9). Moreover, other studies explored the relationship between mobile phone addiction and different levels of shyness (8, 10). These studies did not distinguish the standard by which scores should be regarded as shy individuals and suggested that shyness is a property. Further, they stated that higher scores indicated a higher level of shyness. In this study, we selected the second condition. Individuals with shy properties have numerous disadvantages compared to others. They tend to pick up negative emotions (e.g., loneliness and depression) and are more likely to evaluate themselves negatively (11). Furthermore, shy people usually have decreased social interaction and weaker social ties (12), which could lead to difficulties in life and work. All these negative emotions and real-life difficulties caused by shyness could lead to mobile phone addiction (8). Compensatory Internet Use Theory posits that people turn to the internet or smartphones to escape pain when they encounter psychosocial problems in the real world (13). According to this theory, individuals with a shy property are more likely to relieve their negative emotions and satisfy their need for socialization by using the internet and mobile phones, which provide a social networking environment without face-to-face communication (13, 14). Furthermore, some studies have investigated the relationship between shyness and mobile phone addiction and found that shyness is a potential factor in mobile phone addiction (8, 10, 12). For example, Tian et al. found that problematic mobile phone use could be positively predicted by shyness in a sample of 1,621 undergraduate students (10). Based on the theory and results of empirical studies, this study hypothesizes that mobile phone addiction tendency is significantly and positively predicted by shyness.

Social anxiety may mediate the relationship between shyness and mobile phone addiction. Social anxiety is a common human experience characterized by an intense fear of evaluation from others in social situations (15). Everyone has social needs. Shy individuals also have the motivation to interact with others, but they spend much time monitoring their feelings and behaviors during social interactions, worrying about making a bad impression on others; thus, their normal social needs cannot be satisfied (16). These contradictory results may lead to social anxiety (17). To relieve social anxiety, shy individuals indulge in the online world to satisfy their normal social needs. According to the cognitive model of social phobia (18, 19), individuals with social anxiety generally exhibit negative thinking patterns and are more likely to view neutral social cues as negative signs. This may explain why shy people are vulnerable to anxiety in face-to-face social situations. The internet and mobile phones provide socially anxious people with an ideal tool to alleviate anxiety by creating a less intimate circumstance than that of face-to-face interactions and allowing anxious individuals to escape from personal interactions and immediate responses (20). Research has shown that, compared to others, socially anxious people prefer online social interactions and are more vulnerable to mobile phone addiction (21, 22). For instance, Caplan found that problematic mobile phone use positively correlates with social anxiety (21). Based on the theory and empirical evidence, this study proposes that social anxiety mediates the relationship between shyness and mobile phone addiction.

In addition, the relationship between shyness and mobile phone addiction may be mediated by self-control. Hagger et al. suggest that self-control is an individual's ability to consciously control impulsive behaviors and resist satisfying immediate needs and desires (23). Previous studies have shown that shyness is negatively correlated with self-control (24, 25), and mobile phone addiction can be negatively predicted by self-control (26). Furthermore, Li et al. found that the relationship between loneliness and mobile phone addiction was mediated by self-control. Shyness and loneliness are both negative emotions in nature and should be regarded as predisposing factors to addictive behaviors (26). The reward model of self-control posits that an imbalance between obtaining rewards and exerting effort often leads to decreased self-control (27). On the one hand, individuals with shyness should exert effort to restrain negative emotions in social situations; on the other hand, the outcome of social interaction is often not ideal. The imbalance between the effort they put in and the outcome they received would cause them to have negative emotional problems (28), which leads to decreased self-control. Therefore, this study hypothesized that the relationship between shyness and mobile phone addiction tendency was mediated by self-control.

Furthermore, the relationship between shyness and mobile phone addiction could not only be mediated by social anxiety and self-control but also by them sequentially. As an effective and verified theoretical framework for explaining the development of online addictive behaviors, the Interaction of Person-Affect-Cognition-Execution (I-PACE) model was chosen as the framework for this study. The model explains not only why social anxiety and self-control could mediate the relationship between shyness and mobile phone addiction tendency but also why shyness and mobile phone addiction tendency could be sequentially mediated by social anxiety and self-control. The model posits that addictive behaviors are the result of interactions between predisposing factors, mediators (e.g., affective and cognitive responses), and execution (e.g., coping styles) (29). In this study, shyness was considered a predisposing variable. Social anxiety was included as an affective variable, and self-control was considered a cognitive variable. In this study, shyness was a predisposing variable that could lead to addictive behavior. Social anxiety was considered an affective variable, and self-control was included as a cognitive variable. Specifically, shy individuals have the intention and motivation to communicate with others. However, they spend much time focusing on negative emotions and worrying about making a bad impression on others, leading to social anxiety (17, 30). Self-control can also be affected by social anxiety (8). The limited resources of self-control theory posit that individuals' self-control strength depends on limited resources, and all self-control behaviors (including emotion regulation, mind control, and decision-making) consume the same resources (31). The depletion of self-control resources in some areas leads to a decline in self-control ability (32). One recent study showed that individuals with social anxiety risk poor self-control after social interaction (33). A decline in self-control has been linked to mobile phone addiction (34). Based on the theory of the I-PACE model and empirical evidence, this study hypothesized that the relationship between shyness and mobile phone addiction tendency could be sequentially mediated by social anxiety and self-control.

## The Present Study

**Figure 1:**
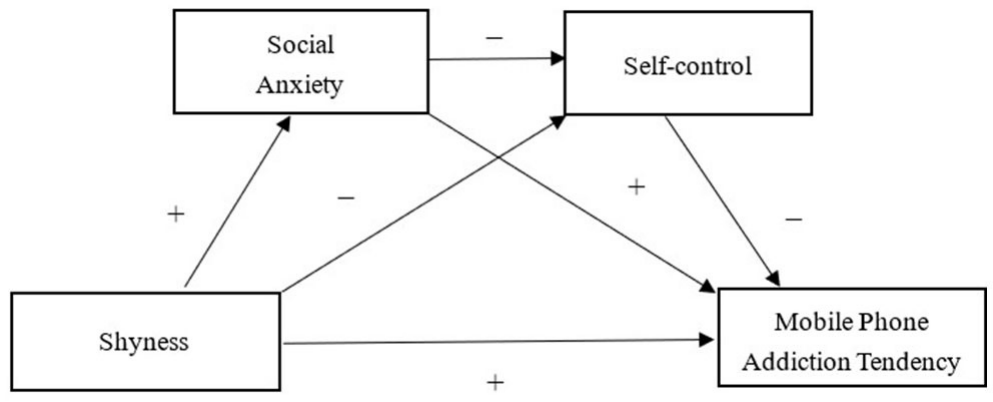
A conceptual model of multiple mediator framework in which social anxiety and self-control mediate the relationship between shyness and mobile phone addiction tendency.

This study aimed to explore the mechanisms underlying the relationship between shyness and mobile phone addiction. Based on these theories and empirical evidence, this study hypothesized that the relationship between shyness and mobile phone addiction could be mediated by social anxiety and self-control in a parallel and sequential manner. The hypothesized model is illustrated in Figure 1. The specific assumptions are as follows.

H1: The tendency to mobile phone addiction was significantly and positively predicted by shyness.

H2: Social anxiety mediates the relationship between shyness and mobile phone addiction tendency.

H3: The relationship between shyness and mobile phone addiction is mediated by self-control.

H4: The relationship between shyness and mobile phone addiction tendency is mediated by social anxiety and self-control sequentially.

## Materials and Methods

### Procedures and Participants

This study used an internet survey to collect data. Before the survey, all participants were informed that the study was conducted anonymously and that their information would be kept confidential. Informed consent was provided in class. The questionnaire link was then sent to participants through the SO JUMP platform, which was used to collect responses and store data. Finally, all of the data were imported into SPSS for further analyses.

The initial sample consisted of 3,606 college students. However, 417 participants who provided incomplete responses were excluded, resulting in 3,189 valid samples for analysis (response rate: 88%), with 1,994 male (62.5%) and 1,195 (37.5%) female respondents. Among them, 1,980 were from the countryside (62.1%). An important inclusion criterion for participant selection was that the mobile phone addiction tendency score was ≥1. These participants were from eight majors, including pedagogy, economics, and business administration. The sample included 1,350 freshmen, 925 sophomores, 531 juniors, and 383 seniors. Their average age was 19 years (SD = 3.70). A total of 1,980 (62.1%) participants were from rural areas, whereas 1,209 (37.9%) were from urban areas. Furthermore, 1,032 (32.4%) participants were from one-child families, whereas 2,157 (67.6%) had siblings. The participants had been using smartphones for an average of 6 years.

### Measures

#### Shyness

Shyness was measured using the revised Cheek and Buss Shyness Scale (35). This tool includes two dimensions (i.e., shyness and sociability) and 13 items (e.g., I am often uncomfortable at parties and other social functions). Respondents were asked to rank their agreement using a five-point Likert scale, namely, “1” = “strongly disagree” and “5” = “strongly agree.” Scores on the 13-item scale ranged from 13 (lowest shyness) to 65 (highest shyness). Higher scores reflected higher levels of shyness. In this study, Cronbach's alpha for the scale was 0.85. The results of CFA showed that χ^2^/*df* = 2.109, CFI = 0.999, AGFI = 0.991, TLI = 0.996, RMSEA = 0.019 (90% CI = 0.012, 0.025), indicating good validity.

#### Social Anxiety

Social anxiety was measured using the short form of social anxiety revised by Wang et al. (36), which includes only one dimension and consists of six items (e.g., “Ignored when in a group”). All items are scored on a 5-point Likert scale (1 = strongly disagree, 5 = strongly disagree). The higher the score, the more severe was the social anxiety. In this study, Cronbach's alpha for the scale was 0.86. The results of the CFA showed χ^2^/*df* = 2.959, CFI = 1.000, AGFI = 0.994, TLI = 0.997, RMSEA = 0.025 (90% CI = 0.000, 0.049), which indicated good validity in this study.

#### Self-Control

This study used the Chinese version of the Self-control Scale (SCS) compiled by Tangney et al. (37) to measure self-control. The scale includes 5 dimensions (i.e., impulse control, healthy habits, resisting temptation, focus on work, and limiting play) and 13 items (e.g., “I could resist temptation very well”). Respondents were asked to rank their agreement on a 5-point Likert scale (1 = not at all, 5 = very much), with higher scores indicating a higher level of self-control. In this study, the internal consistency coefficient of the scale was 0.81. The results of the CFA showed that χ^2^/*df* = 2.863, CFI = 0.998, AGFI = 0.987, TLI = 0.990, RMSEA = 0.024 (90% CI = 0.015, 0.033), indicating good validity in this study.

#### Mobile Phone Addiction Tendency

This study used the MPAI scale designed by Leung (38) to measure mobile phone addiction tendencies. It includes four factors (i.e., Inability to Control Craving, Feeling Anxious & Lost, Withdrawal/Escape, and Productivity Loss) and consists of 17 items (e.g., “You find it difficult to switch off your mobile phone”), with response options ranging from 1 (not at all) to 5 (always). Higher scores indicate a higher level of mobile phone addiction. The internal consistency coefficient of the scale is 0.92. The results of the CFA showed χ^2^/*df* = 3.572, CFI = 0.996, AGFI = 0.980, TLI = 0.988, RMSEA = 0.028 (90% CI = 0.024, 0.033), indicating good validity.

### Statistical Analysis

All analyses were conducted using SPSS 21.0. First, we show the results of the descriptive statistics and then calculate the correlation coefficients among these variables. Second, Model 4 (process macro for SPSS) was used to conduct a mediation analysis of social anxiety and self-control in the relationship between shyness and mobile phone addiction. Third, Model 6 (process macro for SPSS) was used to conduct a sequential mediation analysis of social anxiety and self-control in the relationship between shyness and mobile phone addiction.

### Confirmatory Factor Analysis

We used Mplus 8.0 to conduct a confirmatory factor analysis (CFA) of our focal variables to verify discriminant validity. We compared the hypothesized four-factor model to several alternative models. The results of the chi-square difference test indicated that the four-factor model (Shy, SC, SA, and MP) displayed a significantly better model fit (χ^2^/df = 4.981, RMSEA = 0.035, CFI = 0.951, TLI = 0.946, SRMR = 0.042) than the alternative models (see Table 1), suggesting that our measures had desirable discriminant validity. According to Podsakoff et al., the single-factor model displayed the worst fit, indicating no severe common method biases.

**Table 1:** Confirmatory factor analysis.

**Model**	**χ^2^(*df*)**	**χ^2^*/df***	**Δχ^2^**	**RMSEA**	**CFI**	**TLI**	**SRMR**
4-factor model^a^	5260.00 (1056)	4.981		0.035	0.951	0.946	0.042
3-factor model^b^	7828.00 (1059)	7.391	2568.01***	0.045	0.921	0.913	0.051
2-factor model^c^	9178.66 (1061)	8.651	3918.67***	0.049	0.906	0.895	0.056
1-factor model^d^	11796.38 (1062)	11.108	6536.39***	0.056	0.875	0.862	0.064

*N = 3,189*.

^a^*Hypothesized 4-factor model*.

^b^*Combining SC and SA into one factor*.

^c^*Combining Shy, SC, SA into one factor*.

^d^All factors were combined into a single factor. ***p < 0.001.

## Results

### Common Method Biases

A common variance analysis was applied to the four questionnaires through factor analysis. The chi-square statistic of Bartlett's test of sphericity is significant. After principal component analysis, nine eigenvalues >1 were extracted. The first factor explaining the variance was 31.75%, less than the 40% required by the critical standard (39), indicating that the questionnaires used in this study had no significant common method bias.

### Descriptive Statistics and Correlation Analysis

The descriptive statistics and correlation coefficients among these variables are shown in Table 2. The results showed that mobile phone addiction tendency was significantly and positively correlated with shyness (*r* = 0.50, *p* < 0.01) and social anxiety (*r* = 0.50, *p* < 0.01), and was significantly and negatively correlated with self-control (*r* = −0.57, *p* < 0.01).

**Table 2:** Descriptive statistics and correlation matrix of all variables.

**Variables**	**M**	**SD**	**1**	**2**	**3**	**4**	**5**
Age	19.00	3.70	–				
Shyness	2.47	0.64	0.01	–			
Social anxiety	1.94	0.81	−0.02	0.70**	–		
Self-control	3.35	0.65	0.02	−0.47**	−0.52**	–	
MPAT	2.01	0.71	−0.01	0.50**	0.50**	−0.57**	–

N = 3,189. MPAT, Mobile Phone Addiction Tendency. **p < 0.01.

### Mediating Effect of Social Anxiety

After controlling for age and sex, Model 4 (PROCESS macro for SPSS) was used to test H1 and H2. The results were shown in Table 3 and showed that shyness was positively correlated with social anxiety (*b* = 0.90, *p* < 0.001), and social anxiety was positively related to mobile phone addiction tendency (*b* = 0.25, *p* < 0.001). The residual direct effect was also significant (*b* = 0.34, *p* < 0.001). This result indicates that social anxiety partially mediated the relationship between shyness and mobile phone addiction tendency (indirect effect = 0.22, 95% CI = 0.18–0.26), thereby supporting H2. This model accounted for 39.3% of the variance in mobile phone addiction.

**Table 3:** Results of mediation analysis of social anxiety.

	**M: SA**			**Y: MPAT**		
	***B***	***SE***	**95%*CI***	***B***	***SE***	**95%*CI***
X: shyness	0.90***	0.02	0.86, 0.93	0.34***	0.02	0.29, 0.38
M: SA	–	–	–	0.25***	0.02	0.21, 0.28
age	−0.01	0.02	−0.04, 0.04	−0.01	0.00	−0.01, 0.00
gender	−0.01	0.02	−0.04,0.04	−0.01	0.02	−0.05,0.04
Constant	−0.17*	0.07	−0.32, −0.03	0.71***	0.08	0.57,0.86
*R^2^* = 0.49				*R^2^* = 0.29		
*F*_(3, 3, 185)_ =1036.99***				*F*_(4, 3, 184)_ = 480.22***		

SA, Social Anxiety; MPAT, Mobile Phone Addiction Tendency. ^***^p < 0.001.

### Mediating Effect of Self-Control

H3 was tested using the PROCESS macro for SPSS (Model 4) after controlling for age and sex. The results were shown in Table 4 and showed self-control was negatively predicted by shyness (*b* = −0.48, *p* < 0.001), and mobile phone addiction tendency was negatively predicted by self-control (*b* = −0.47, *p* < 0.001). The residual direct effect was also significant (*b* = 0.33, *p* < 0.001). Thus, self-control also played a partial mediating role in the link between shyness and mobile phone addiction tendency (indirect effect = 0.23, 95% CI = 0.21–0.25), supporting H3. This model accounted for 41.1% of the variance in mobile phone addiction.

**Table 4:** Results of mediation analysis of self-control.

	**M: SC**			**Y: MPAT**		
	***B***	***SE***	**95%*CI***	***B***	***SE***	**95%*CI***
X: shyness	−0.48***	0.02	−0.51, −0.45	0.33***	0.02	0.29, 0.37
M: SC	–	–	–	−0.47***	0.02	−0.51, −0.44
age	0.00	0.00	0.00,0.01	0.00	0.00	−0.01,0.01
gender	−0.01	0.02	−0.05,0.03	−0.01	0.02	−0.05,0.03
Constant	4.54***	0.05	4.44, 4.63	2.77***	0.10	2.58, 2.96
*R^2^* = 0.22				*R^2^* = 0.40		
*F*_(3, 3, 185)_ = 304.02***				*F*_(4, 3, 184)_ = 525.50***		

SC, Self-control; MPAT, Mobile Phone Addiction Tendency. ***p < 0.001.

### Multiple Mediation Model

The results of multiple mediation analysis of social anxiety and self-control were shown in Table 5. After controlling for age and sex, Model 6 (PROCESS macro for SPSS) was used to test the multiple mediation model. The results showed the pathways for “shyness → social anxiety → mobile phone addiction tendency” (indirect effect = 0.10, 95% CI = 0.06 0.13). The pathways for “shyness → self-control → mobile phone addiction tendency” (indirect effect = 0.08, 95% CI = 0.07 to 0.10) were significant. These results indicate that social anxiety and self-control mediated the relationship between shyness and mobile phone addiction. The sequential pathway for “shyness → social anxiety → self-control → mobile phone addiction tendency” was significant (indirect effect = 0.10, 95% CI = 0.09–0.12). Furthermore, the residual direct effect was significant (*b* = 0.24, *p* < 0.001). This result indicates that social anxiety and self-control partially mediated the relationship between shyness and mobile phone addiction tendency. This multiple mediation model explained a significant portion of the variation in mobile phone addiction tendencies (*R*^2^ = 0.41).

**Table 5:** Testing the pathways of the multiple mediation model.

**Effect**	***B***	***SE***	**95%CI**	
**Direct effects**
Shyness → social anxiety	0.90***	0.02	0.86	0.93
Shyness → self-control	−0.21***	0.02	−0.26	−0.17
Social anxiety → self-control	−0.30***	0.02	−0.33	−0.27
Shyness → mobile phone addiction tendency	0.24***	0.02	0.20	0.29
Social anxiety → mobile phone addiction tendency	0.12***	0.02	0.08	0.15
Self-control → mobile phone addiction tendency	−0.43***	0.02	−0.47	−0.40
**Indirect effects**				
Shyness → social anxiety → mobile phone addcition tendency	0.10	0.02	0.06	0.13
Shyness → self-control → mobile phone addcition tendency	0.08	0.01	0.07	0.10
Shyness → social anxiety → self-control → mobile phone addiction tendency	0.10	0.01	0.09	0.12

N = 3,189. ***p < 0.001.

## Discussion

This study found that mobile phone addiction tendency among college students could be positively predicted by shyness, supporting H1. The present finding was consistent with previous studies (10, 11). For example, the research conducted by Tian et al. found that shyness positively influences generalized pathological internet use. One possible explanation is that the internet provides shy people with a communication environment that satisfies their social needs without the anxiety and discomfort associated with face-to-face communication (8).

This study found that social anxiety partially mediated the relationship between shyness and mobile phone addiction tendency, thereby supporting H2. The present finding was similar to previous studies (2, 40). One explanation for this result could be that shyness makes individuals addicted to mobile phones through social anxiety. Shyness is a form of social withdrawal (41). Social motivation theory divides social withdrawal into three subtypes: shyness, unsociability, and social avoidance (42). Compared to individuals with unsociability and social avoidance, shy individuals have both high social approach motivation and social avoidance motivation, which results in the greatest psychological avoidance conflict among the three social withdrawal subtypes. On the one hand, shy individuals have a strong desire to communicate with others, but on the other hand, they often feel nervous in the face of communication. These contradictory results may lead to social anxiety. Another explanation could be that shy individuals cannot obtain an identity, leading to social anxiety. Adolescence is a critical period for acquiring identity (43). However, shy individuals have difficulties expressing themselves and understanding others, belonging to a group, and being approved by a group. Difficulties in acquiring identity may result in social anxiety. Many studies have shown that shyness is often associated with negative consequences of social adaptation (44). Shy individuals are prone to developing problems, such as low self-esteem and social anxiety (45). Shy individuals are more prone to using mobile phones to relieve social anxiety and satisfy normal social needs.

Furthermore, this study found that the relationship between shyness and mobile phone addiction tendency was mediated by self-control, thereby supporting H3. This finding is consistent with that of previous studies (2, 24). For instance, Han et al. found that low self-control mediated the impact of shyness on mobile phone addiction. One possible explanation is that shyness weakens self-control, which, in turn, leads to mobile phone addiction. According to the limited resource model of self-control (46), activities, such as emotional control, may lead to reduced self-control, resulting in problematic behaviors (47). Shy individuals are typically introverted and nervous. They usually adopt chronic and negative coping strategies, such as withdrawal and escapism, which lower self-control levels (48). Research has shown that low self-control levels lead to various behavioral problems and addictions (49, 50), including mobile phone addiction (51). According to theory and empirical evidence, the relationship between shyness and addiction to mobile phones was mediated by self-control.

The main result of this study was that social anxiety and self-control mediated the relationship between shyness and mobile phone addiction. This result was similar to that of previous studies (24, 40), which could be explained by the I-PACE model (29). This result suggests that shy individuals often experience high social anxiety levels that consume cognitive resources, thus impairing self-control (46) and eventually increasing the risk of mobile phone addiction (52). The I-PACE model posits that the occurrence and development of addictive behaviors result from predisposing variables, affective and cognitive responses to specific stimuli, and executive function (29). In this study, shyness was a predisposing factor for mobile phone addiction (14), impairing individuals' affective and cognitive activities. In this study, individuals with shyness experienced social anxiety because they could not meet their normal social needs (45). Whereas, this negative feeling would consume resources used to control behavior, thus impairing their self-control and eventually leading to mobile phone addiction. The results of the multiple mediation model, therefore, support important explanatory mechanisms in which predisposing factors (e.g., shyness) could impair individuals' affective (social anxiety) and cognitive activity (self-control). The results of this study were conducted under the guidance of the I-PACE model, and the results supported the I-PACE model.

## Implications for Practice

This study has both theoretical and practical implications. Theoretically, this study not only enriches related research in this field but also supports the I-PACE model, which posits that addictive behaviors are the consequence of predisposing variables (e.g., shyness), affective (e.g., social anxiety), and cognitive (self-control) responses to specific stimuli and executive functions. Practically, this study analyzed why shyness could affect mobile phone addiction tendencies. On the other hand, this study also provided some ways (i.e., alleviating social anxiety and strengthening self-control) to alleviate mobile phone addiction among shy college students. To help shy college students avoid the negative influence of mobile phone addiction, educators can offer courses on interpersonal communication to teach them how to communicate with others and reduce their social anxiety. Educators can also help shy college students eliminate mobile phone addiction by strengthening self-control. Self-control training can improve self-awareness and self-monitoring (53, 54), which is beneficial for reducing addictive behavior (55, 56).

## Limitations and Future Directions

Some limitations of this study should be noted. First, it used a cross-sectional design, which limited the exploration of the causal relationship among these variables. Second, we did not perform a good sampling job. We chose convenient sampling, which could lead to systematic errors and make it difficult to generalize our research results. Therefore, future research should focus on participant sampling. Third, although this study investigated the mechanisms underlying the relationship between shyness and mobile phone addiction, we did not consider moderating variables that could mitigate the negative effect of shyness. These limitations should be addressed in future studies.

## Conclusion

This study explored the mechanism (i.e., the mediating roles of social anxiety and self-control) underlying the relationship between shyness and mobile phone addiction tendency based on a sample of 3,189 Chinese college students. The results showed that social anxiety and self-control mediated the effect of shyness on mobile phone addiction tendency in a parallel and sequential manner. These results discuss some implications for helping shy college students overcome mobile phone addiction (e.g., alleviating social anxiety and strengthening self-control).

## Data Availability Statement

The raw data supporting the conclusions of this article will be made available by the authors, without undue reservation.

## Ethics Statement

The studies involving human participants were reviewed and approved by Zhejiang Normal University. The patients/participants provided their written informed consent to participate in this study.

## Author Contributions

Conceptualization: WX and WL. Methodology and writing—original draft preparation: XL. Validation: ML and HZ. Resources: WL. Writing—review and editing: XL and WX. Supervision: ML, WL, and HZ. All authors contributed to the article and approved the submitted version.

## Funding

This project was supported by Open Research Fund of College of Teacher Education, Zhejiang Normal University (No. jykf22040) and Party Building and Ideological and Political Education Research Institute, Zhejiang Business College (No. SZY_SZ202210).

## Conflict of Interest

The authors declare that the research was conducted in the absence of any commercial or financial relationships that could be construed as a potential conflict of interest.

## Publisher's Note

All claims expressed in this article are solely those of the authors and do not necessarily represent those of their affiliated organizations, or those of the publisher, the editors and the reviewers. Any product that may be evaluated in this article, or claim that may be made by its manufacturer, is not guaranteed or endorsed by the publisher.
